# The “*facie sympathique”* sign in hanging: historical background, forensic review, and perspectives

**DOI:** 10.1007/s12024-023-00603-8

**Published:** 2023-03-09

**Authors:** Daniela Marchetti, Luca Santoro, Giulia Mercuri

**Affiliations:** 1 Department of Health Care Surveillance and Bioethics, Section of Legal Medicine, Università Cattolica del S. Cuore, L.go F. Vito 1, 00168 Rome, Italy; 2 Fondazione Policlinico Universitario A. Gemelli IRCCS, L.go F. Vito 1, 00168 Rome, Italy

**Keywords:** Forensic pathology, Oculo-sympathetic paresis, *Facie sympathique*, Neck compression, Hanging

## Abstract

The “*facie* *sympathique*” is a vital sign first described by Etienne Martin in 1899 referring to unilateral miosis, with or without ptosis, at the opposite side from the knot in hanging. This mark is scarcely reported in legal medicine textbooks and scientific papers. Moreover, when cited, it is referred to differently from its original meaning, both as unilateral contraction (miosis) and dilatation (mydriasis) of the pupil depending on the antemortem firmness of the ligature’s neck pressure in hanging with little attention to ptosis. Due to the sympathetic nervous pathway supplying the eye, the review of this ocular sign in hanging supports the importance of revitalizing the “*facie sympathique*” in research on lesion vitality in mechanical asphyxia.

## Introduction

Hanging is the most frequent form of mechanical asphyxia in suicide in most countries [1, 2]. However, the judicial authority does not frequently pursue investigations by forensic autopsy and toxicological examination in hanging cases. In Italy, for instance, the magistrate usually limits the forensic investigation to medico-legal doctor participation at the crime scene and external examination. Hence, the medico-legal contribution does not reduce the risk of a misclassified death cause and manner. Forensic researchers have long been engaged in distinguishing antemortem from postmortem hanging biomarkers through the analysis of skin wounds at the ligature neck mark [3], fractures of the neck structures [4–6], injuries of the soft tissue [7], and the presence of intimal layer tears in the carotid artery (Amussat’s sign) and hemorrhages on the anterior surface of the intervertebral lumbar disc (Simon’s bleedings) [8–10].

**Fig. 1:**
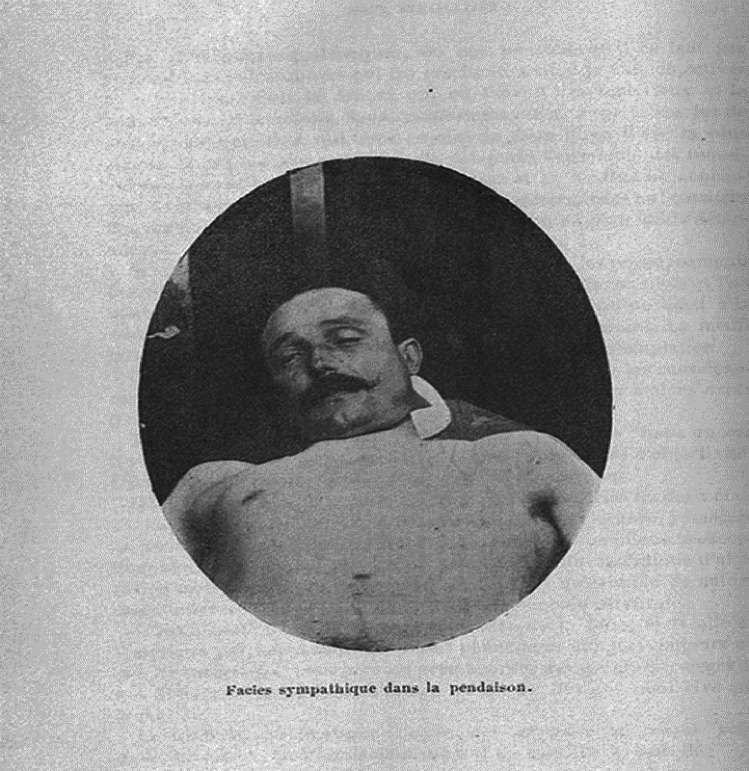
Photograph of the “*facie sympathique*” in an individual who died due to hanging. Etienne M. “Le facies sympathique des pendus.” Archives de l’anthropologie criminelle (vol. 14; 1899, Page 178), Musée Criminocorpus consulted on Nov. 9, 2022, https://criminocorpus.org/en/ref/114/9565/

In 1899, Etienne Martin (1871–1949) first described the presence of unilateral miosis with partial superior eyelid closure (ptosis) on the opposite side of the knot in two individuals who died due to hanging (Fig. 1), which he named the “*facie sympathique”* as it resembled a patient’s face after sympathectomy. However, disagreement about the interpretation of the “*facie sympathique*” as an antemortem ocular mark of neck compression arose soon after its first description (1899) [11, 12].

This paper offers a historical and narrative medico-legal contribution to better understand whether the “*facie sympathique*” finding should be re-evaluated by forensic pathologists as a marker of vital compression of the neck, a key forensic analysis topic in cases of mechanical asphyxia caused by hanging.

## Historical background

An important initial aspect is a revision of the studies on hanging conducted by Etienne Martin (1871–1949) and Nicolae Minovici (Bucarest, 1868–1941) at the beginning of the twentieth century.

Etienne Martin, who followed A. Lacassagne as the chair of forensic medicine in 1913 at the *Médecine Légale Institute* of the *Faculté de Médecine de l’Université de Lyon,* reported two cases of hanging where the loop of the rope was deeper at the left side of the neck (first case) and at the right side (second case) in an article titled “*Le facies sympathique des pendus*” (1899) [11]. In the first case, he observed a concomitant presence of miosis (5 mm on the left vs. 7 mm on the right) and ptosis in the left eye. He also noted tearing of the intimal layer of the left carotid artery (Amussat’s sign) and hemorrhage of the ipsilateral superior sympathetic ganglion at the noose level. In the second case, he noted miosis at the right pupil, facial congestion, tongue placement between the teeth, and hemorrhagic petechiae at the legs.

In both cases, in the opinion of Etienne Martin, the facial findings resembled those observed by the surgeon M. Jaboulay in patients sympathectomized for exophthalmos due to goiter [13]. Thus, based on surgical knowledge, Etienne Martin suggested that compression and/or stimulation by stretching of the cervical sympathetic trunk is responsible for pupillary inequality in hanging.

It could be argued that the close cooperation between French and Romanian researchers was the reason why in 1904, Nicolae Minovici (of Bucarest) visited the laboratory of Etienne Martin at the *Médecine Légale Institute* of the *l’Université de Lyon* to “*faire la connaisence*” [14]. In the book “*Studiu asupra spânzurării”* [15], translated into French in 1905, Nicolae Minovici reported his experience on 51 cases, considering his results as “*absolutament contraries*” to those of Etienne Martin; it was assumed that the higher-strength neck compression was at the opposite side of the knot (loop) [14]. Although Nicolae Minovici did not provide any alternative pathogenic explanation to Etienne Martin’s thesis, he stated that pupillary and eyelid inequality represents an unpredictable postmortem facial sign that can be associated with individuals who died from disease rather than from hanging. However, some of the data collected by Nicolae Minovici (Table 1) do not seem to be “*absolutament contraries*” from Etienne Martin’s experience. By reviewing the information in Table 1, Minovici described miosis at the side of the loop in 5 of 51 hanging cases (case n.19 at left, n.15 at right; n.6, 7, 8 loop anteriorly) and ptosis at the side of the loop in 1 of 51 cases (case n.6 on the right side). Moreover, Minovici reported pupilar “*inégales*” on the same side of the loop in 7 of 51 cases (n.9, 10, 11, 12, 16, 17, 20).

**Table 1:** Autopsy data collected by Minovici [14, 15]

**Knot to the right side of the neck (left strong neck compression)**					**Knot to the left side of the neck (right strong neck compression)**					**Occipital knot (anterior strong neck compression)**					**Anterior knot, under the chin (occipital strong neck compression)**				
**OS**			**OD**		**OS**			**OD**		**OS**			**OD**		**OS**			**OD**	
**N. of case**	**Pupil**	**Eyelid**	**Pupil**	**Eyelid**	**N. of case**	**Pupil**	**Eyelid**	**Pupil**	**Eyelid**	**N. of case**	**Pupil**	**Eyelid**	**Pupil**	**Eyelid**	**N. case**	**Pupil**	**Eyelid**	**Pupil**	**Eyelid**
**1**	O	^—^	O	^—^	**1**	O	^—^	O	^—^	**1**	O	^—^	O	^—^	**1**	O	^⌢^	O	^⌢^
**2**	O	^—^	O	^—^	**2**	O	^—^	O	^—^	**2**	O*	^—^	○	^—^					
**3**	O	^—^	O	^—^	**3**	O	^—^	O	^—^	**3**	O	^⌢^	○	^—^					
**4**	O	^—^	O	^—^	**4**	O	^—^	O	^—^	**4**	O	^⌢^	O	^⌢^					
**5**	O	^—^	O	^—^	**5**	O	^—^	O	^⌢^	**5**	O	^⌢^	O	^⌢^					
**6**	O	^—^	O	^—^	**6**	NR	^⌢^	NR	^—^	**6**	●	NR	●	NR					
**7**	O	^—^	O	^—^	**7**	O	^⌢^	O	^⌢^	**7**	●	NR	●	NR					
**8**	O	^—^	O	^—^	**8**	O	^⌢^	O	^⌢^	**8**	●	NR	●	NR					
**9**	O*	^—^	○	^—^	**9**	O	^⌢^	O	^⌢^	**9**	●	NR	O	NR					
**10**	O*	^—^	○	^—^	**10**	O	^⌢^	O	^⌢^	**10**	O	NR	O	NR					
**11**	O*	^—^	○	^—^	**11**	O	^⌢^	O	^⌢^	**11**	O	NR	O	NR					
**12**	○*	^—^	O	^—^	**12**	O*	^⌢^	O	^⌢^	**12**	O	NR	O	NR					
**13**	O	^⌢^	O	^⌢^	**13**	O*	^⌢^	O	^⌢^										
**14**	O	^⌢^	O	^⌢^	**14**	O*	^⌢^	O	^⌢^										
**15**	O	^⌢^	O	^⌢^	**15**	O*	NR	●	NR										
**16**	○*	^⌢^	O	^⌢^	**16**	O	NR	O	NR										
**17**	○*	^⌢^	O	^⌢^	**17**	O	NR	O	NR										
**18**	O*	^⌢^	O	^⌢^	**18**	O*	NR	O	NR										
**19**	●	NR	O	NR															
**20**	O*	NR	O	NR															

*OD* oculus dexter, *OS* oculus sinister, miosis ●, mydriasis O, nondilated pupil ○, ptosis ^—^,  open eyelid ^⌢^, pupil inequality *, *NR* not reported

## Medico-legal narrative review

Many legal medicine textbooks and scientific papers have been published since the beginning of the twentieth century. However, assessments of this peculiar facial finding have rarely been conducted in the international medico-legal literature [16]. Nevertheless, the presence of anisocoria in hanging is cited as a “significant” central nervous system sign [17] or as a “specific” sign [18]. Payne-James et al. reported that anisocoria, although rarely observed in hanging, “may be caused by pressure of the strangulation device on the cervical sympathetic nerve” [19]. Interestingly, when these ocular signs are mentioned, they are considered vital signs and reported differently from Etienne Martin’s original experience regarding the presence of miosis, the involvement of the upper eyelid (ptosis), and the site of the neck at which the rope’s section (knot or loop) is believed to be responsible for the pressure on cervical sympathetic nerves. Several authors described the “*facie sympathique*” as mydriasis and an open eyelid at the side of the knot instead of miosis and partial eyelid closure (ptosis) at the side of the loop, as described by Etienne Martin [20–23]. Polson et al. described the presence of anisocoria on the side of the ligature knot [24] without specifying the eye signs (miosis or mydriasis). In the *Forensic Atlas* edited by Weimann and Prokop [25], the presence of ptosis is described in asphyxia deaths related to strangulation while eyelids are not visible in the pictures regarding hanging. The pupil status is not shown in both cases. Finally, most of the forensic case reports on hanging retrieved by searching the PubMed database up to September 2022 have the victim’s eyes hidden by a black strip for privacy.

**Fig. 2:**
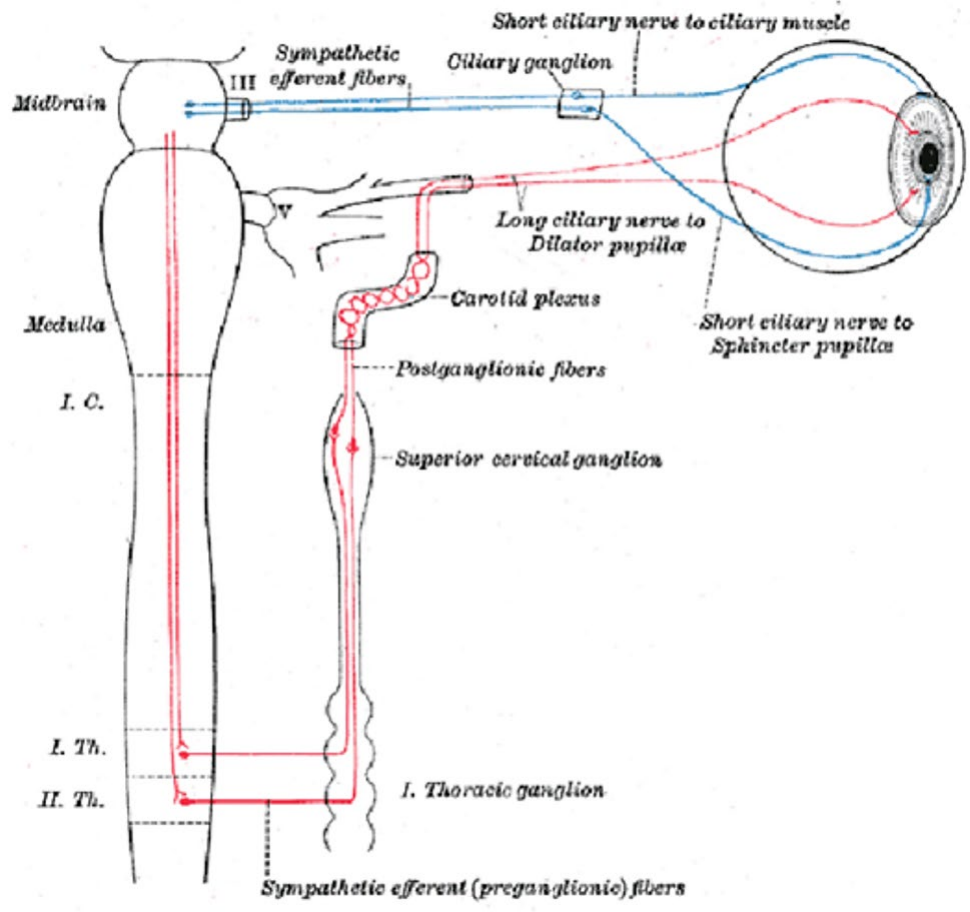
Anatomy of the sympathetic nervous system supplying the eye. Gray, Henry. Anatomy of the Human Body. Philadelphia: Lea & Febiger, 1918; Fig. 840, Sympathetic connections of the ciliary and superior cervical ganglia. Bartleby.com, 2000, consulted on Nov. 9, 2022. www.bartleby.com/107/

The differences in “*facie sympathique*” reporting in hanging among forensic texts might have an historical explanation. Undoubtedly, the interruption of the cervical sympathetic nervous system supplying the eye (Fig. 2) with unilateral miosis together with ptosis dates to 1852 in the report of Claude Bernard [26], a researcher at the *Faculté de Médecine de l’Université de Lyon* (the same institution as Etienne Martin). However, evidence of ocular inequality due to sympathetic involvement was previously identified in dogs by Pourfour du Petit (1775), who gave his name to the syndrome characterized by unilateral mydriasis (not miosis), eyelid retraction (not ptosis), and sometimes hyperhidrosis [27]. These ocular signs (mydriasis and eyelid retraction) have been considered by recent researchers [28] due to an interesting lesion (not an interruption) observed in the cervical sympathetic nervous system and may precede Claude Bernard’s disease.

Thus, there are two ways that involvement of the cervical sympathetic nervous system might produce the “*facie sympathique*” in hanging: nerve interruption at the site of the ligature’s strongest compression, causing miosis, or nerve excitation by stretching (not interruption), which might occur in the area close to the point of the knot, causing mydriasis.

## Medico-legal perspectives

The idea of a relationship between the “*facie sympathique*” (miosis and ptosis) and cervical sympathetic pathway damage in hanging does not seem to be confined to the beginning of the twentieth century. Nevertheless, the antemortem anatomic involvement of the cervical sympathetic tract in hanging did not garner substantial forensic attention over time. The scarcity of “*facie sympathique*” reporting could be because it is believed that only very strong neck pressure might injure the cervical sympathetic chain and because unilateral miosis might be considered a postmortem behavior of the pupil [29], although unusual and not well understood. Thus, whether the “*facie sympathique*” is due to an antemortem cervical sympathetic neck injury or if it represents a postmortem change in pupil diameter undoubtedly requires further research.

It would be intriguing for forensic investigation to concentrate on eyelid and iris details with the same attention usually paid to other hanging marks. This crude observational activity, hopefully with the help of a measuring tool to avoid subjective results, could be first used to evaluate whether, as per Etienne Martin, the term “*facie sympathique”* must be maintained (or not) in association with neck compression in hanging or, as per Minovici, it must be considered an unpredictable and unstable ocular mark. Moreover, processes for diagnosing Bernard-Horner syndrome in living people [30], postmortem computer tomography (CT) or nuclear magnetic resonance (NMR) [31], could be promising tools for detecting the integrity of the superior cervical ganglion prior to autopsy [32], although access to both of these techniques remains limited in forensic institutes. Finally, in living people, anatomical cervical sympathetic integrity is also investigated by the chemical response of the pupils to pharmacological tests [30, 33]. The use of these pharmacological tests postmortem has not been considered, although parameters of pupil reaction are used to establish the time since death through topical drugs that act directly on iris muscles, with divergent results [34–41] (Table 2) regarding the mechanism of this supravital phenomenon.

**Table 2:** Postmortem chemical smooth iris muscle excitability

**References**	**N. of cases**	**Mydriatic agent (concentration)**	**Myotic agent (concentration)**	**Postmortem iris excitability (h)**		**Site of injection**	**Method of measuring pupil size**
Marshall [34]	15	Atropine (N.R)		1.20 − 4		A.C C.S	Scale enclosed in Nettleship’s pocket
			Pilocarpina (N.R)		0.20		
			Eserina* (N.R)		0.5		
			Ergotina (N.R)		0.5 − 2		
Prokop and Fϋnfhausen [35]	n.r	Homatropine (0.1%)		8 − 17		A.C	NR
			Pilocarpine (0.1%)		14 − > 20		
Bardzik [36]	50	Atropine (1%)		20		A.C	Zeiss Pupillometer
		Adrenaline (0.1%)		N.R			
			Prostigmine* (0.5%)		> 20		
			Pilocarpine (2%)		20		
Klein and Klein [37]	3979	Tropicamide (0.25%)			5 − 30	A.C C.S	N.R
		NOR/adrenaline (1%)			14 − 46		
		Atropine Ciclopentolate (1%/0.5%)			3 − 10		
			Acetylcoline (5%)		14 − 46		
Orrico et al. [38]	309	Atropine (NR)			0 − 26	A.C C.S	Pupillometer
			Pilocarpine (N.R)				
			Atro-Pilo (N.R)				
Larpkrajang et al. [39]	100		Pilocarpine (2%)		1.22 − 17.6		Vernier Caliper
Koehler et al. [40]	137	Tropicamide (NR)			< 5	C.S	Digital-Photography + Pupil-Iris Ratio
			Acetylcholine (N.R)		5 min to 14		
Shivam et al. [41]	200		Pilocarpine (2%)		0.5	C.S	ImageJ Freewar

*A.C* anterior chamber injection, *C.S* conjunctival sac instillation, *NR* not reported, *indirect-acting agonists with respect to acetylcholinesterase

## Conclusion

Undoubtedly, only a careful evaluation of all the available data may reduce the risk of a misinterpretation of death’s cause and manner, especially in the asphyxia-related deaths. Anyway, what can be drawn from the aforementioned data? First, forensic investigators have maintained an interest in “*facie sympathique*” in hanging over time, although this mark is described differently from Etienne Martin’s description (1899) without further evidence regarding antemortem versus postmortem pathogenetic mechanisms. Hence, the historical disagreement between Etienne Martin and Nicolae Minovici regarding the interpretation of the “*facie sympathique*” has not been overcome, and this facial mark cannot yet be recommended as a diagnostic tool. For this reason, the opportunity to appreciate ocular inequality is of forensic interest, and the “*facie sympathique*” mark should be a focus of studies aimed at identifying signs of vitality in hanging and in any fatal upper neck trauma, especially when no skin marks are evident.

## Key points

1. Forensic researchers have long distinguished antemortem from postmortem hanging markers.
2. In 1899, Etienne Martin first noted unilateral miosis with partial superior eyelid closure (ptosis) on the opposite side of the knot in hanging. He named this presentation “*facie sympathique*” due to its resemblance to the face of patients who underwent sympathectomy.
3. This facial mark is scarcely reported in the forensic literature and, when mentioned, is described differently from its original meaning. Thus, the “*facie sympathique*” cannot be recommended as a diagnostic tool in establishing antemortem from postmortem neck compression.
4. For these reasons, the evidence of ocular inequality in hanging might maintain forensic interest and allow the consideration of the “*facie sympathique*” mark in fields of research focused on signs of vitality in hanging and any fatal upper neck trauma, especially when no skin marks are evident.

## Data Availability

The authors confirm that the data supporting the findings of this study are available within the article and/or from the corresponding author.
